# Surgical Resection of Vascular Anomalies of the Upper Extremity—An Observational Study †

**DOI:** 10.3390/jcm14061930

**Published:** 2025-03-13

**Authors:** Christina Scharitzer, Florian Wolf, Caspar Wiener, Thomas Rath, Martin Metzelder, Christine Radtke, Eva Placheta-Györi

**Affiliations:** 1 Department of Plastic, Reconstructive and Aesthetic Surgery, Medical University of Vienna, Spitalgasse 23, 1090 Vienna, Austriachristine.radtke@meduniwien.ac.at (C.R.); 2 Department of Paediatric and Adolescent Surgery, Medical University of Vienna, 1090 Vienna, Austria; florian.wolf@meduniwien.ac.at; 3 Department of Biomedical Imaging and Image-Guided Therapy, Medical University of Vienna, 1090 Vienna, Austria; caspar.wiener@meduniwien.ac.at (C.W.); martin.metzelder@meduniwien.ac.at (M.M.)

**Keywords:** haemangioma, venous malformation, arteriovenous malformation, lymphatic malformation, hand, pain

## Abstract

**Background/Objectives**: This study aimed to investigate surgical resections of vascular malformations and haemangiomas of the upper extremity, pre- and postoperative symptoms, complications and recurrences. **Methods**: A total of 82 patients with vascular malformations and haemangiomas treated by surgical resection from 2010 to 2020 were included in this observational study. Pre- and postoperative symptoms, including pain and swelling, as well as complications and recurrence rates, were assessed. Descriptive statistics were provided for all reported data. Parametric and non-parametric tests were used for group comparisons. Alterations of reported pain were assessed. A two-sided alpha of 5% determined statistical significance. **Results:** A total of 88 procedures were performed in 82 patients. The most common vascular malformation was a venous malformation, followed by arterio-venous malformations. More than 50% of the patients reported pain prior to the surgery, while 14.6% of patients experienced pain postoperatively, which indicated significant improvement (*p* = 0.001). Minor postoperative complications occurred in 31.7% of patients. Overall, a recurrence rate of 17.1% was recorded during postoperative follow-up, mostly occurring in diffuse and infiltrating types of vascular malformations. **Conclusions**: Surgical resections of vascular anomalies of the upper extremity led to symptom improvement and are an important part of the multidisciplinary treatment algorithm.

## 1. Introduction

Vascular malformations occur in 1.5% of the general population [1]. Venous malformations are the most frequent malformation and make up 70% of vascular malformations [2,3]. The most common locations of vascular malformations are the head and neck followed by the extremities [4].

Although vascular malformations and vascular tumours such as haemangiomas are benign growths, they may prove difficult to treat due to their location, extent or involvement of critical anatomical structures. Symptoms range from asymptomatic to impairing due to pain, swelling or pulsation, and can even cause a loss of function in the affected extremity in rare cases. This looks to be especially problematic in the upper extremity due to the importance of function [5,6].

Vascular malformations were first classified in 1982 by Mulliken and Glowachi [7]. In 1996, this classification was adapted by the International Society for the Study of Vascular Anomalies (ISSVA) and revised in 2014 [8]. It divided them into proliferating vascular lesions (tumours) and vascular malformations [9]. Vascular malformations can be classified based on their vessel of origin or hemodynamic characteristics into low-flow (venous, lymphatic, capillary, combined malformations) and high-flow malformations (arterio-venous, fistulas) [8,10,11,12]. Due to the broad presentation of vascular malformations, treatments decisions are met on a case-to-case basis [13,14]. Treatment options include conservative treatments (observation, compression therapy and physical therapy), interventional therapy (sclerotherapy, embolisation and laser therapy) and surgical resection [15,16,17]. For selected malformations, pharmacological options such as mTOR inhibitors and beta-blockers can be used as treatment [1,18,19]. The type of treatment necessary depends on lesion characteristics such as size, location, structures affected and clinical presentation and progression over time [20,21]. Surgical resection can either be a step of a multimodal treatment plan or the only treatment modality in smaller localised lesions [19,22]. Complex vascular malformations involving vital structures pose treatment challenges that exceed the limitations of surgical therapy [23].

This study aims to examine the role of surgical resection of vascular malformations and haemangiomas in the upper extremity, given its distinct functional significance.

## 2. Materials and Methods

Patients who underwent surgical resections of vascular malformations and haemangioma of the upper extremity from 2010 to 2020 were included in this retrospective single-centre observational study. All included cases were seen by the interdisciplinary Vascular Anomaly Board at the university hospital. Patients presenting with vascular anomalies not affecting the upper extremities, as well as those not surgically treated, were excluded from this analysis. Ethical approval for this study (Reference No. 1097/2021) was provided by the institutional Ethics Committee on 16 March 2021. The study was registered with the Research Registry database (https://www.researchregistry.com/, UIN 10382, accessed on 10 June 2024).

Medical histories and surgical reports were collected using the hospitals digital database. Parameters assessed included patient demographics, type of malformation (arterio-venous malformations, venous malformations, lymphatic malformations, capillary malformations and haemangiomas), size and location of the malformation. Preoperative symptoms (pain, swelling, ulceration, elevated temperature and pulsating sensation) and postoperative progress and pain were analysed. The type and number of operative treatments, length of hospital stay and follow-up time were assessed. Postoperative complications (minor complication healing under conservative treatment; major complications requiring surgical revision [24]) were recorded and postoperative recurrence rated analysed.

All included patients signed a photo release statement and consented to scientific analysis and publication.

Statistical Analysis: All data were reported according to the PROCESS criteria [25]. Descriptive statistics including mean value, standard deviation and range were reported for all patient data. Parametric and non-parametric tests were used for group comparisons depending on the normal distribution of the data. Clinical symptoms such as swelling, ulceration, elevated temperature and a pulsating sensation were presented using frequency tables. The pain before and after the operation was cross-tabulated and the McNemar test was carried out to determine that the procedure improved the patient’s reported pain. The patient collective was grouped and analysed based on the type of vascular malformation. A two-sided alpha of 5% was applied in all statistical tests. Programs used for the statistical analysis and graphs included SPSS Statistics (Version 25, IBM©), Excel (Version 2310, Microsoft 365©) and GraphPad Prism 9 (Version 9.2.0).

## 3. Results

A total of 82 patients were included in this study, with 59 female and 23 male patients (Table 1). A total of 26 were children and 56 adults. The average age was 30.2 ± 19.7 years (range: 0 to 80 years). The age distribution of the high-flow and low-flow subgroups was found to be congruent (*p* = 0.02).

A total of 65.9% had low-flow and 34.1% of patients high-flow vascular malformations, including 10 patients (12.2%) of the population with haemangiomas. The most common types of vascular anomalies were venous malformations with a total of 47 (57.3%)—of these, 13 were male—followed by 18 arterio-venous malformations (22%), including 4 male patients. (Table 1). There was no significant difference in the occurrence of high-flow or low-flow vascular malformations in either men or women (*p* = 0.55).

Most vascular malformations were located on the digits (27.4%), and the forearm (16.7%). There was an even distribution of malformations located on the left and on the right half of the body (Table 2).

Clinical characteristics, including pain, swelling, ulceration, elevated temperature and pulsating sensation, are described in Table 3 for each subgroup.

**Figure 1:**
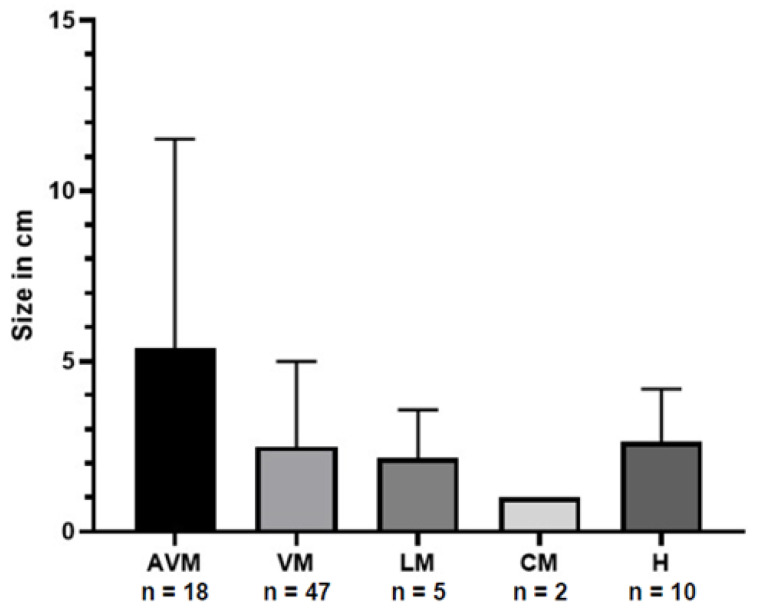
Distribution of the size of vascular anomaly in centimetres (bars represent the standard deviation) grouped by type of malformation. AVM = arterio-venous malformation; VM = venous malformation; LM = lymphatic malformation; CM = capillary malformation; H = haemangioma.

The size of the vascular malformations varied from 0.2 cm to 25 cm (mean: 3.0 cm, SD: 3.5 cm). The size (in centimetres) of the vascular malformations and haemangiomas grouped by the type of vascular anomaly is shown in Figure 1.

**Figure 2:**
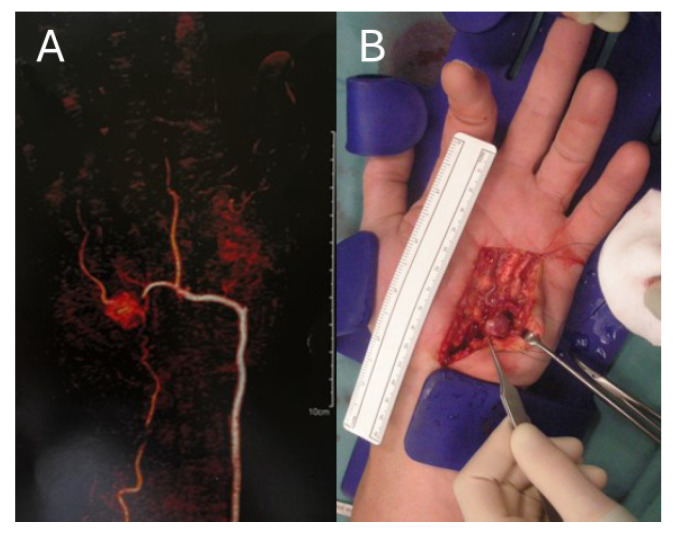
Left hand of a 62-year-old male with an arterio-venous malformation in the hypothenar region; (**A**) CT-angiography with a contrasting agent which shows an arterio-venous malformation of the left hypothenar; (**B**) arterio-venous malformation during surgical resection.

A total of 88.6% (*n* = 78) of vascular anomalies of the upper extremities were completely resected (Figure 2), while one partial resection and three amputations were executed.

One partial resection of a venous malformation on the left thenar was performed in a six-year-old female patient due to the infiltrative nature of the malformation to avoid mutilation of the hand function. The procedure was carried out using neuromonitoring and the continuity and function of the affected median nerve branches were preserved.

Preservation of the main vessels was possible in all cases where the vascular anomalies were resected. Therefore, no vascular reconstruction was required and the perfusion of the upper extremity was not endangered in any of the included patients.

In the analysed patient group, three amputations were performed. Two of the patients who underwent an amputation suffered from an extensive arterio-venous malformations. One patient presented with a recurring ulcerating venous malformation. This was an 80-year-old female patient who underwent an amputation of the distal phalanx of her fourth digit on the left hand, which was functionally severely impaired.

A three-year-old male patient underwent amputation of his right hand due to severe pain, swelling, ulceration and functional impairment caused by an extensive arteriovenous malformation. The lesion was 10 cm in diameter on the right palm and extended to phalanges one to four. The indication to amputate was made by a multidisciplinary committee after four sclerotherapy sessions did not result in symptom control. The patient’s hand function was severely impaired by swelling and progression of the arterio-venous malformation.

A 53-year-old female patient underwent an amputation of her right forearm, which was affected by a complex arterio-venous malformation leading to pain, ulceration, discoloration, and a chronic contracture resulting in severe functional impairment.

In all 82 patients, the average length of hospital stays was 3.7 days (range: 1 to 8 days). The minimum stay was 1 day, which included outpatient treatments.

There were no intraoperative complications in the analysed patient population. Postoperatively, 26 (31.7%) minor complications and 1 major complication occurred (Table 4). Minor complications included minor hematoma, localised wound infection, delayed wound healing, paraesthesia and swelling that healed conservatively. The incidence of postoperative complications differed by the type of vascular anomaly. The highest rate of postoperative complications was in the group of lymphatic malformations, with 3 out of 5 patients affected (Table 4). Results show no significant correlation between the occurrence of a complication and high- or low-flow of the vascular anomalies (*p* = 0.38).

Fourteen (17.1%) patients presented with local recurrences of the vascular malformation. The recurrence rate grouped by type of vascular anomaly is shown in Table 2. The recurrence rate in the venous malformations group was 19.1%. In one patient with capillary malformations and two patients with lymphatic malformations, recurrences appeared. A comparison of high- and low-flow malformations and recurrence was carried out. Results show no significant cohesion between the two (*p* = 0.12).

**Figure 3:**
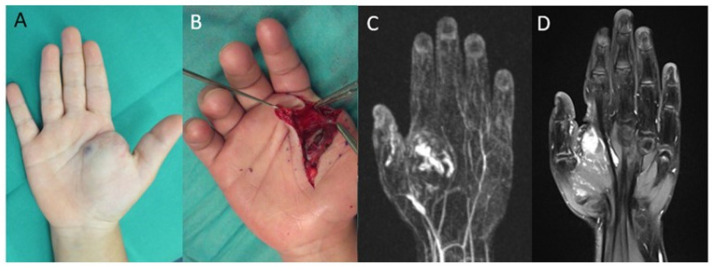
Right hand of a five-year-old female patient with an arterio-venous malformation of the right hand with diffuse intermuscular invasion: (**A**) blue discoloration and swelling in the palm and thenar region of the right hand; (**B**) intraoperative image of the arterio-venous malformation; (**C**) contrast-enhanced MR-angiography shows an arterio-venous malformation of the palm and thenar region of the right hand; (**D**) MRI of a recurrence of the arterio-venous malformation 6 years after surgical resection.

Out of 14 patients, 2 required sclerotherapy and 6 needed a second surgical resection. The remaining patients were treated conservatively or further treatment was not necessary. A patient example of a recurrent arterio-venous malformation is shown in Figure 3.

The mean postoperative follow-up was 24.9 weeks (range: 1 day–335.1 weeks; SD: 56.3 weeks). Two patients were lost to follow-up.

As documented in the medical history, 56.1% of patients reported pain prior to the surgical procedure, while 30.5% reported no pain. In 13.4%, a pain sensation was not documented; henceforth, it is rated as no pain. Postoperatively, the rate of patients reporting pain was reduced to 14.6%. This reduction was statistically highly significant (*p* = 0.001).

**Figure 4:**
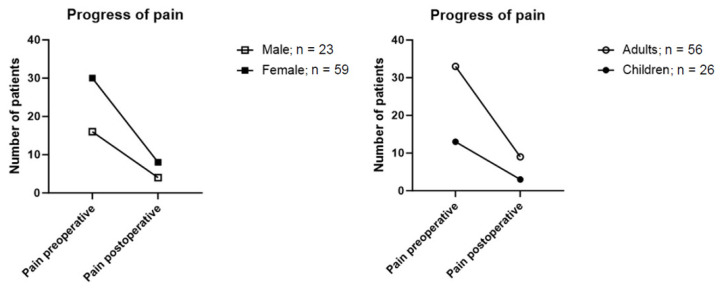
Reported pain preoperatively and after surgical resection grouped by sex and age.

Subgroup-analyses comparing age and gender, as well as paediatric and adult patients, were conducted (Figure 4). Male participants reported higher rates of preoperative pain at 69.6% (*n* = 16) compared to females at 50.8% (*n* = 30). Preoperative pain was reported by 58.9% of adults (*n* = 33) and 50% of children (*n* = 13). The subgroup most affected by pain before surgery was the venous malformation group, that reported pain in 63.8% of cases (30 out of 47 patients).

In postoperative follow-up, 17.4% (*n* = 4) of male and 13.6% (*n* = 8) of female patients reported persistence of pain after the operation. A subgroup analysis based on age distribution showed a total of 13% (*n* = 3) of children and 16.1% (*n* = 9) of adults still reported postoperative pain (Figure 4).

## 4. Discussion

In this observational study, we analysed the surgical treatment of vascular anomalies in the upper extremity. Treating vascular malformations of the upper extremity is challenging due to the complexity of anatomical structures and their functional importance [26]. Complex cases are discussed in a multidisciplinary board including plastic surgeons, paediatric surgeons, radiologists and vascular surgeons [13,27,28,29,30]. Treatment options such as sclerotherapy and embolisation, which can cause tissue necrosis, pain, systemic complications and nerve injury, as well as pharmacological therapy, which is limited depending on the cellular activity of the vascular malformation, are discussed as additional or alternative treatment to surgical resections [31,32,33]. This study focused on patients undergoing surgical resections to specifically analyse the postoperative course of treatment, complication rates and symptom development.

The present study has a relatively large study population (*n* = 82) overall, compared to similar studies [34,35,36], with a higher number of female patients included (ratio male:female = 1:2). This lies in contrast to other studies, where no gender difference was reported [5].

Vascular malformations are divided into groups according to their flow dynamics and vascular component (ISSVA classification) [8,9,37]. In unison with other studies, we found venous malformations to be the most common subgroup, followed by arterio-venous, lymphatic and capillary malformations [2,5,15].

In alignment with previous reports, vascular malformations of the upper extremity were most commonly located on the hand [38]. As they can cause pain, ulceration, physical impairment, deformation and may enlarge through the influence of hormones or injury, it is especially important to treat them [4,39]. Pain at the site of the vascular malformation was one of the most important characteristics, affecting over 50% of patients. A core finding of this study was the significant improvement of the reported pain after surgical resection of the vascular anomalies. Laurian and colleagues also showed significant improvements of pain in patients with venous malformations of the forearm after surgical resection [34].

In our study, around 30 percent of patients presented with a minor postoperative complication that was managed conservatively. A difference between the occurrence of complications in high-flow versus low-flow malformations was not determined. In a recent systematic review and meta-analysis, minor complications after surgical resection ranged from 0% to 42% in arterio-venous, 0% to 12% in venous and 0% to 17% in lymphatic malformations [40].

Recurrences of vascular malformations are often attributed to the diffuse and infiltrative nature of certain types of malformations, as well as lesion progression triggered by factors such as trauma or hormonal changes [23]. Liu and colleagues found a recurrence rate of 81% in patients with arterio-venous malformations who had undergone surgical resection [23]. Most studies included in a systematic review showed recurrence rates ranging from 0 to 14% [40]. In the present study, 17.1% of patients presented with a local recurrence. Differences concerning the number of recurrences between high- and low-flow malformations were not found. In a recent meta-analysis, it is emphasised that complete resection of vascular malformations reduced recurrence risks [40].

Study limitations include the retrospective design, multiple subgroups due to the heterogeneous nature of vascular anomalies and limited postoperative follow-up. This study did not focus on the comparison of surgical treatment to sclerotherapy and embolisation. Future studies ought to prospectively assess multiple treatment modalities and include the patient’s perspective and quality of life.

## 5. Conclusions

Surgical resection of vascular malformations of the upper extremity is an important part of the treatment algorithm in the multidisciplinary approach required for comprehensive patient management. The data showed that the pain levels after the surgical resection improved significantly in patients where operative treatment was indicated.

## Figures and Tables

**Table 1:** Clinical presentation of patient demographics and characteristics of vascular anomalies in the upper extremity.

	AVM *n* = 18 (22%)	VM *n* = 47 (57.3%)	LM *n* = 5 (6%)	CM *n* = 2 (2.4%)	H *n* = 10 (12.2%)	Total *n* = 82 (100%)
**Demographics**						
Female	14 (78%)	34 (72%)	3 (60%)	2 (100%)	6 (60%)	59 (72%)
Male	4 (22%)	13 (28%)	2 (40%)		4 (40%)	23 (28%)
Age in years	39.5 ± 23.3	29.9 ± 17.7	15.2 ± 13.6	26.5 ± 13.4	22.8 ± 20.3	30.2 ± 19.7
Size in cm ± SD	5.6 ± 6.3	2.4 ± 2.4	2.2 ± 1.4	n/a	2.6 ± 1.6	3.0 ± 3.5

Nominal data are presented as absolute numbers (*n*) and percentage (%). Metric data are presented as mean values ± standard deviation. AVM, arterio-venous malformation; VM, venous malformation; LM, lymphatic malformation; CM, capillary malformation; H, haemangiomas; n/a: not applicable.

**Table 2:** Clinical presentation of the location of vascular anomalies on the upper extremity.

	AVM *n* = 18 (22%)	VM *n* = 47 (57.3%)	LM *n* = 5 (6%)	CM *n* = 2 (2.4%)	H *n* = 10 (12.2%)	Total *n* = 82 (100%)
**Location of Malformation**						
Digits	7 (8.3%)	11 (13.2%)	2 (2.4%)	1 (1.2%)	1 (1.2%)	23 (27.4%)
Palm	3 (3.7%)	7 (8.3%)	2 (2.4%)		1 (1.2%)	13 (15.5%)
Dorsal hand	1 (1.2%)	1 (1.2%)			2 (2.4%)	5 (6.0%)
Wrist	1 (1.2%)	7 (8.3%)			1 (1.2%)	10 (11.9%)
Forearm	3 (3.7%)	8 (9.8%)	1 (1.2%)	1 (1.2%)	1 (1.2%)	14 (16.7%)
Elbow	1 (1.2%)	4 (4.8%)			1 (1.2%)	7 (8.3%)
Upper arm	1 (1.2%)	3 (3.7%)			1 (1.2%)	5 (6.0%)
Axilla		2 (2.4%)				2 (2.4%)
Shoulder		3 (3.7%)			2 (2.4%)	2 (2.4%)
Multiple locations	1 (1.2%)	1 (1.2%)				2 (2.4%)

Nominal data are presented as absolute numbers (*n*) and percentage (%). Metric data are presented as mean values ± standard deviation. AVM, arterio-venous malformation; VM, venous malformation; LM, lymphatic malformation; CM, capillary malformation; H, haemangiomas.

**Table 3:** Clinical presentation of characteristics of vascular anomalies in the upper extremity.

	AVM *n* = 18 (22%)	VM *n* = 47 (57.3%)	LM *n* = 5 (6%)	CM *n* = 2 (2.4%)	H *n* = 10 (12.2%)	Total *n* = 82 (100%)
**Preoperative Symptoms**						
Pain	8 (44.4%)	30 (63.8%)	2 (40%)	1 (50%)	5 (50%)	46 (56.1%)
Swelling	5 (27.8%)	18 (39.1%)	3 (60%)	1 (50%)	3 (30%)	30 (36.6%)
Ulceration	3 (16.7%)	1 (4.7%)	1 (20%)			5 (6.1%)
Elevated Temperature and Pulsating Sensation	4 (22.2%)					4 (4.9%)

Nominal data are presented as absolute numbers (*n*) and percentage (%). Metric data are presented as mean values ± standard deviation. AVM, arterio-venous malformation; VM, venous malformation; LM, lymphatic malformation; CM, capillary malformation; H, haemangiomas.

**Table 4:** Clinical presentation of complications and recurrences after surgical resection separated by type of malformation.

	AVM *n* = 18 (22%)	VM *n* = 47 (57%)	LM *n* = 5 (6%)	CM *n* = 2 (2.4%)	H *n* = 10 (12%)	Total *n* = 82 (100%)
**Complications**						
Minor	7 (38.9%)	11 (23.4%)	3 (60%)	1 (50%)	4 (40%)	26 (31.7%)
Major	0	1 (2.1%)	0	0	0	1 (1.2%)
**Recurrences**	1 (5.5%)	9 (19.1%)	2 (40%)	1 (50%)	1 (10%)	14 (17.1%)

AVM, arterio-venous malformation; VM, venous malformation; LM, lymphatic malformation; CM, capillary malformation; H, haemangiomas.

## Data Availability

The datasets used during the current study are available from to corresponding author upon request.
